# The use of home care as relational work: outlines for a research programme

**DOI:** 10.1080/17482631.2024.2371538

**Published:** 2024-06-24

**Authors:** Tove Harnett, Glenn Möllergren, Håkan Jönson

**Affiliations:** School of Social Work, Lund University, Lund, Sweden

**Keywords:** Eldercare, home care, care theory, care users, emotion work, relational work

## Abstract

**Purpose:**

Care has been theorized as a relational practice, but the research has focused on providers rather than users. Older care users have been cast in a passive role, and their relational activities to help with the provision of their care or to support those who provide it are underexplored. The purpose of this study is to develop knowledge about home care use as a form of relational ‘work’.

**Methods:**

The data for the study consists of 34 qualitative interviews with home care users in Sweden and 15 observations of care provision. The data has been coded using thematic analysis.

**Results:**

The analysis identifies two overlapping forms of relational work done by care users in the home care context: care-centred work, where care users work to facilitate care situations that were positive for staff and for the provision of care; and person-centred work, where care users work to foster personal relations by focusing on care staff as unique individuals.

**Conclusions:**

The article proposes a research programme on relational work by care users, prompted by the finding that such efforts seem central for the understanding of eldercare in a variety of contexts.

## Introduction

In an interview about the provision of home care, the respondent, Anette, talked about ways to create a relaxed atmosphere during intimate care. Her strategy was to use chitchat and humour to defuse the situation, adapting her behaviour to each individual. Remarkably, Anette was not a care provider but a 94-year-old care recipient – or, in this article, a *care user* – sharing her tactics for putting care staff at their ease. Some care staff seemed nervous and embarrassed when helping her in the shower and she used personalized strategies to facilitate a smoother provision of home care services. Her actions and her stated reasons are strikingly similar to an approach developed in theories of care and nursing.

Theories of care have evolved within different traditions, but they all share the notion that care is a relational practice (Tronto, 1993; Van Dijke et al., 2020). Gilligan (1993) emphasized empathy, compassion, and emotional connection in care, noting its gendered aspects. Wærness (1984) coined the term ‘the rationality of caring’ to shed light on how caregivers apply their own understanding based on relations, moral responsibility, and an empathetic attitude towards the shifting needs of those who receive care. Nordic care researchers, such as Davies (1994), Eliasson-Lappalainen (2005), and Szebehely (1995, 1998) further developed this approach for formal eldercare, studying social class and the organization of care as part of the Scandinavian welfare system. The care theory approach holds that good care cannot be standardized in the kinds of procedures that characterize industrial work: not only do people’s needs vary, but moral responsibility demands a focus on the particular character of care situations. Hochschild (2012) has identified care as emotion work and emotional labour and her theory has been further developed in models on emotion management in care (Cranford & Miller, 2013). Andersson et al. (2023) use the term ‘empathetic attuning’ to highlight formal carers’ working skills. A similar emphasis on relational aspects and emotion is present in theories of nursing and person-centred care (Dewar & Nolan, 2013; Fazio et al., 2018; Nolan et al., 2006; Pejner et al., 2012), and empathy has been theorized and studied as a core concept in care ethics (Van Dijke et al., 2019, 2020). There is a considerable literature on person-centred care that highlights the need to acknowledge the care recipient as a unique person, build trustful relations, and include the individual in decision-making processes (Ebrahimi et al., 2021; Fazio et al., 2018).

Care theory holds the dyadic caregiver–care receiver relationship to be the crucial element in understanding care. This approach offers valuable insights into family caregivers’ moral reasoning, especially in challenging conditions (Ulmanen, 2013; Wallroth, 2016). Similarly, several studies have looked at the precariousness of formal carers in different care regimes (Fine, 2015; Schwiter et al., 2018; Theobald, 2011), using a care theory approach to highlight the moral obligations that care workers feel they have, and their strategies for providing good care under difficult circumstances (Daly & Szebehely, 2012; Hirvonen & Husso, 2012; Martin-Matthews, 2007; Strandell, 2022).

However, as Fine (2015) has noted, most explorations of the actors’ ‘doings’ in these relationships have been one-sided, leaving the person who receives – or uses – the care out of the picture. The role of the care user has been acknowledged in some versions of care theory (Tronto, 1993; Van Dijke et al., 2019, 2020), but relations performed among care users have rarely been theorized as an effort or accomplishment – a form of relational ‘work’. Several studies have investigated the experience of older care users, but older people have primarily been cast as *affected* by care (Doyle, 2014; Fjordside & Morville, 2016). Studies of older people’s views portray their well-being and dignity as being ‘maintained’ by professionals, overlooking the care users’ own actions (Hemberg et al., 2020; Larsson et al., 2023; Norell et al., 2012; Pejner et al., 2012).

There are some studies of older people which speak to their active participation in eldercare (Lewinter, 2003; Olaison et al., 2021; Palmqvist, 2020). Kittay (2011), for example, argues that the term ‘care’ denotes labour that requires skills on the part of the caregiver and uptake by the care user, while Ahnlund et al. (2023) note that a ‘good working relation for both parties’ is central for home care provision and use. Cranford and Miller (2013) show how care users signal their unmet needs to care workers as a way of steering activities. Yet care users’ relational activities designed to facilitate the provision of care and support those who provide it – as empathizers and sympathizers – are underexplored and insufficiently theorized. As 94-year-old Anette’s case shows, care users may adopt an empathetic, supportive demeanour to facilitate their care provision, expressing sympathy and support for staff who they think are experiencing difficulties. Anette’s approach could be described in terms of a ‘rationality of caring’ (Wærness, 1984) or as a case of reciprocal action within care relations (Lewinter, 2003).

The present study investigates care as a relational practice, but shifts the focus from care providers to care users. What relational activities are performed by care users, what are their reasons, and what do they think they will achieve? *The purpose of the study is to develop knowledge about home care use as a form of relational work, with components that older people consider and apply with varying degrees of skill and energy*. Inspired by care theory and the sociology of emotions, the emphasis is on the empathetic understanding and sentiment demonstrated by many care users when interacting with care staff, and the findings are related to the ‘working conditions’ of older people in Sweden’s home care system. Our investigation is limited to formal care. While focusing on the Swedish context, we would argue that care users in other countries are engaged in similar forms of relational work, which could be analysed in terms of the organizational constraints and regimes of any given context.

## Formal home care in Sweden

Sweden’s tradition of providing formal care for older citizens in their own homes dates to the early 1950s (Szebehely & Trydegård, 2012). Home care is the backbone of eldercare, with about 250,000 users (Socialstyrelsen, 2022b). Home care services depend on the outcome of a needs assessment; when eligible, the applicant receives a formal decision about a set of services, which are provided by a care organization – local authority, non-profit, or for-profit – according to a purchaser–provider system. The provision of services is predominantly publicly funded but care users pay a fee. The fee is never more than 220 Euro a month (in 2024), even for those receiving home care as often as eight or ten times a day. Care users with low incomes pay less or nothing. Services range from help with personal hygiene, taking medication, and getting dressed to cleaning, laundry, social support, and outdoor walks. Swedish home care has increasingly been subject to cutbacks, marketization, and a new public management-inspired assembly line logic (Erlandsson & Szebehely, 2023; Szebehely & Meagher, 2018), where service provision is characterized by minutely planned intervention schedules, a stressful work environment, and large staff turnover with widespread discontent among care workers. Care users must navigate the welfare system, including home care services and greater assistance from private companies, transportation services, healthcare, and other agents of the welfare state. Accessing nursing home care has become increasingly challenging, leaving some home care users to rely on help multiple times daily. On average, Swedish home care users will meet 16 different staff members in a fortnight (Kolada, 2023). Despite these challenge, user surveys consistently shows high satisfaction levels among users (Socialstyrelsen, 2022a).

## Method

The data for the study was collected as part of a larger research project about Swedish home care users. The project concerns symbolic interactionism and centres on the way individuals interpret and act on their understanding of events in specific contexts, using a reflexive process that includes interaction with oneself and others (Blumer, 1986). The design of the study follows Bradshaw et al. (2017), who stress that qualitative description research is a tool with which to ‘discover and understand a phenomenon’, especially ‘where information is required directly from those experiencing the phenomenon under investigation’ (1). The data consists of 34 qualitative interviews with 36 care users aged between 68 and 96 – 22 women and 14 men – in southern Sweden and 15 observations of home care provision in 6 care users’ homes. Interviews were carried out between 2021 and 2023 in respondents’ homes, a variety of settings ranging from the centre and suburbs of Sweden’s third-largest city to small towns and rural areas. Among the respondents were retired blue-collar workers, farmers, academics, and business owners. Four had been born outside Sweden. A majority lived in single households, but in five instances a cohabiting partner contributed to answering the interview questions, either partially or entirely, in collaboration with or in the presence of the care user. Participants had varying care needs, but most had home care several times a day. There was also variation in their care experience, ranging from a few months to more than 20 years.

Most respondents were recruited through the home care organizations of three local authorities, one of which was engaged in a pilot study that informed the main investigation (and included in the data for the present study). Recruitment was boosted by media coverage, a social media presence, and an announcement on the university department’s website, which encouraged interested individuals to make contact with the research team.

This study uses multiple methods and a pragmatic approach in the tradition of qualitative research using thematic analysis to understand home care use in ordinary life. The design of the study followed the approach developed by Blumer (1986), with an open exploration phase followed by an inspection phase with a more specific focus. The interview guide was drafted using a pilot study of 11 participants, all of whom were included in the sample. The pilot study interviews were characterized by a conversational tone, using broad and open-ended questions. The research team’s analysis of the pilot study data was carried out in discussion. The guide included questions about respondents’ experiences using home care and their views on what constitutes good and bad care. Interviews lasted between 30 and 120 minutes and several questions focused specifically on relationships:

- How would you describe relations between you and the care staff? Do they differ depending on which staff member you have in mind?
- Have relations between you and the care staff changed over time?
- Do you sometimes talk to staff members about other things that the actual service provision, such as family or other personal matters?
- What is your opinion about talking about personal matters, such as family members or events in your life?

Participant observations during home care was the second type of data. Recruitment included the information process for interviewees: based on an evaluation that included ethical concerns, some were asked if they were interested in participating in the observation study. Convenience sampling was used so there were at least five participants and a variety of situations to study. The observations were designed to verify and nuance findings and visualize aspects of the interviews. While the interview guide covered some of our initial assumptions, the observations were an opportunity to experience the care situation in a more unfiltered manner, capturing ordinary interactions that had escaped the interviewees’ notice, so they had not commented on them in their interview responses. The researcher arrived at respondents’ homes approximately 20 minutes before the home care staff was supposed to arrive and remained throughout the service provision process, before (when the care users were asked about their expectations), during, and after (when care users were interviewed again once staff had left). The care-related situations include getting out of bed, having breakfast, preparing meals, taking medication, cleaning, and showering. An observation guide was used and detailed fieldnotes were taken. The observer focused on greetings, instructions, and the interactions as care was provided, and especially small talk, jokes, and talk about their private lives. All observations were followed by a short reflective interview (audio recorded and transcribed) when the care users could comment on the meaning of the interactions which had occurred. This data was also part of the analysis. Unlike other studies (Fry et al., 2013), we did not attempt to decide the character of emotional activities based on our own interpretations of body language, eye contact, or conversational tone during the observations, although such aspects were documented and discussed in the follow-up interviews once staff had left. In presenting the findings, we have only included observations that care users commented on during follow-up interviews.

All interviews were audio-recorded and transcribed verbatim. The process of analysis consisted of steps that initially followed the principles of thematic analysis (Braun & Clarke, 2006) but introduced theory in the later stages of the analysis. During a first step, we read through interview transcripts and fieldnotes in order to familiarize ourselves with data and identify ideas. Data was closely reviewed to generate codes and the dataset was coded in order to identify themes relating to the overarching topic on relations. Codes were refined and added during this stage of the analysis. During the next step, concepts from care theory and from the sociology of emotion (Clark, 1997; Hochschild, 2012) guided the analysis in identifying activities, techniques, and reasons for the actions that respondents outlined. Findings were continually discussed among the members of the research team to increase the study’s reliability.

In the analysis an important distinction is made between empathy and sympathy. Empathy is the emotional ability to understand the feelings and situation of 'the other' – the empathee – and recent studies of care ethics have stressed empathy’s relational character (Van Dijke et al., 2019, 2020). Sympathy adds sentiment to empathy (Clark, 1997). When regarded as ‘emotion work’ (Hochschild, 2012), empathy and sympathy appear as deep-acting in cases when feelings are congruent with their expression, and surface-acting when emotions and display are disconnected. A typical example of the latter would be when a care user praises the work of care staff, despite being dissatisfied.

The study was approved by the Swedish Ethical Review Authority (Dnr 2022–00829–02). Each participant was informed about the study’s objectives and the possibility to withdraw consent at any stage. Particular attention was paid to participant observations, with continuous discussions during visits to determine which aspects the respondents wanted researchers to observe. All participants have been anonymized.

## Results

The analysis identified two overlapping forms of relational work that care users carried out in a home care context: *care-centred work* and *person-centred work*. Care-centred work facilitates care situations that were positive for staff and for care provision. When engaged in this form of work, users addressed staff as professionals in their roles as home care workers. Two forms of care-centred work were identified: (1) boosting staff and (2) protecting staff. Person-centred work, meanwhile, refers to care users’ efforts to foster personal relations by focusing on staff as unique individuals. Three forms of person-centred work were identified: (1) adjusting communication styles and topics, (2) showing an interest in the person, and (3) finding common ground.

Emotional engagement is central to both care-centred work and person-centred work, manifested as empathy about the situation of the staff and single individuals. This work was also expressed as feelings of sympathy, love, fondness, and connectedness. Care users often described a self-directed mission to take an empathetic approach when engaging with staff, related to beliefs and skills that the care user claimed to have because of their particular background, and to staff ‘sympathy accounts’ (Clark, 1997) as individuals or a group.

### Care-centred work

Care-centred work is a type of relational activity designed to make working conditions easy for staff, the care user, or both. It was achieved by boosting staff in various ways, including cheerful greetings, praise (‘You’re the best!’ or ‘I give you five stars’), and positive reinforcement focusing on what staff had done well. Care-centred work also took the shape of protecting staff from negative experiences, for example by helping them avoid unpleasant tasks or criticism.

### Boosting staff

Respondents described a range of ways to boost staff and so improve their working situation and make it more pleasant. Boosting could consist of affirmative greetings (the commonest), praising and encouraging staff, or applying positive ratings. During an observation, a staff member who visited Charlotte (woman, 83) was greeted with particular warmth because she was recently back from sick leave. During the visit, Charlotte said the staff member was one of her ‘favourites’ and she was happy she was working again. After the staff member had left, Charlotte confirmed that her appreciative comments were sincere. Her example highlights the dual character of positive greetings as felt and displayed (Hochschild, 2012), an emotional congruency which was prominent in our data.

Another strategy seen during one participant observation was adopted by Lena (woman, 89), who said goodbye to staff with the exclamation ‘Five stars!’, the highest rating on her own five-point scale. In the follow-up interview, Lena said it was her way of making staff feel that they had done well. The routine could be problematic when she felt things had been done badly, and she explained how she tried to cover up the embarrassing absence of her highest grade by pretending to cough or by going to the bathroom just as staff were leaving and might expect a ‘grade’. Apart from avoiding situational embarrassment and ‘emotive dissonance’ (Hochschild, 2012), these techniques made it possible to maintain the ostensible sincerity of the grading system as such.

Praise for the staff’s excellent work was common in our data, and some users communicated this during the visits we observed. One form of care-centred work involved positive reinforcement, when care users concentrated on what was working well and tried to be forgiving when problems arose. Praise seemed part of a strategy to achieve care situations that were positive for all involved, as illustrated by Janet (woman, 77):

To show one’s appreciation and that, I also often do that to them, that I never argue with anyone or so, scold or criticise straight away like that. But I like to praise them when I think they have done something that is … that I am happy when I see that it [a sticking plaster] is applied so nicely, yes, it feels good.

Care users shared experiences of making complaints and instances when they had chosen not to. To ‘show one’s appreciation’, not ‘criticize straight away’, or ‘give in a bit to keep the atmosphere happy’ were depicted as techniques to make the situation better for staff. Several respondents suggested they were good at handling people – meaning care staff – because of their professional background or personality, and these characteristics were also understood as a useful basis for empathy. The same Janet as quoted earlier backed up her position on positive critique by referring to her own background as a tour guide, and described by contrast a situation where a friend had lost her temper and acted disrespectfully towards a home care worker. It was evident that approaches differed. Some care users reacted to perceived shortcomings in their care in a direct manner and such confrontations resulted in a tense situation, as outlined in conversations and observed when care was performed.

‘Sympathy sentiment’, according to Clark (1997), is ‘the counterpart in one person of another’s sense of discomfort, loss, sorrow, and the like’. A frequently mentioned cause for sympathy among care users we interviewed was what they saw of the home care workers’ stressful working conditions. Ebba (woman, 82), stated that the staff that visited her ‘deserve more appreciation’, and added ‘I feel sorry for them because they are only human’. This form of sympathy could also concern specific categories of staff, as when Andreas (man, 78) discussed those of foreign descent who struggled with the Swedish language: ‘It’s not their fault, but it’s a pity for them that they don’t understand’. A language barrier usually presented as negatively affecting care users was in this case framed as the staff members suffering.

### Protecting staff

Efforts to protect staff stemmed from the same type of sympathy and took the form of being helpful, facilitating work processes, and helping home care workers save time. Users mentioned various ways of helping staff carry out tasks, such as preparing for visits, asking their own relatives for extra help, declining services they had been granted, and pretending not to notice when staff were in their homes for less time than scheduled. These efforts made it possible to avoid conflicts and embarrassment. Several respondents mentioned occasions where they had exposed attempts by staff to ‘cheat’, and the shame and conflict associated with such confrontations. Complaints risked eroding relationships, so users bore in mind not only their own needs, but also those of staff, as illustrated by expressions such as ‘you have to see it from both sides’, ‘cooperate’, and ‘help them a little’. Acting calm and positive, despite feeling upset and dissatisfied, was a recurring form of emotion management that was part of the repertoire of care-centred efforts.

An empathetic approach was central when care users reflected on their own bodies being problematic for staff. This was evident in conversations about women being helped to shower by young men. It is common for female care users to request female staff when showering or for intimate care (Twigg, 2003). The request has traditionally been easy for care providers to comply with since the majority of the home care staff in Sweden are women. There is a growing number of male care workers of foreign origin, however, and several respondents spoke of receiving care from young men predominantly from Muslim countries. Gina (woman, 93), invoked a third party to declare her position on being helped to shower by young men from Muslim countries.

People can say: how do you feel about young guys, especially from Arab countries, from the Middle East, coming here and washing your behind and stuff like that? Then I say: ‘if they … if they do it well, it’s all right’, but I wonder; how do they feel about it? Has anyone thought about how they feel? It may not be pleasant for them at all, but they have to do it. It is not just how I feel, it is important that they feel good. So I can ask: ‘are you … do you feel comfortable with it?’

Gina related her approach to her long career as a teacher and her engagement in social issues. Concerns about embarrassing staff were shared by several respondents. Janet’s comments highlighted one such worry: the potential perception of an older female body as repulsive, which she feared might discomfort staff.

So I have to think, yes, but how do they feel? And it’s quite intimate, because I have a catheter, I have a stoma, it’s very close to body stuff. And they may not be used to it, to see, especially not women, naked or only on the body parts, how they perceive it. It might be a shock for them. And especially an old body perhaps. That you have to take into account how they are, how they feel. (Janet)

Janet elaborated on the ‘two sides’ of the topic, and the phrase ‘So I have to think’ could be regarded as her instructing herself how to handle the situation when younger staff provided intimate care. Twigg (2003) has related such concerns to the vulnerable position of the older care user. What our analysis highlights is that these concerns also have other meanings. In conversation, Andreas used the phrase ‘you can see it, it’s difficult for them’ and ‘it’s not fun for them’ when commenting on the help he needed after visiting the bathroom, but added that it was something that the staff still had to learn how to handle. He thus justified the provision of his intimate care with a considerate reference to the development of a professional approach among staff.

In interviews, many respondents justified their actions by referring to what they thought or knew care staff would feel. Anette joked and chatted because ‘you relax as a helper’ when the care user seemed relaxed; Gina and Janet invoked age and gender, and took religion into consideration when worrying about the need to protect staff.

### Person-centred work

The risk of reducing people to categories has been extensively discussed in care work studies, which has resulted in approaches that start from the position that each person is a unique individual (Fazio et al., 2018; McCormack, 2004). The opposite is true if care staff are viewed as interchangeable – mere hands and legs to serve the care user. The point of person-centred work by care users is to make home care workers feel unique as human beings. These activities could improve the care situation, but in our data were predominantly efforts to acknowledge staff as individuals.

### Adjusting communication styles and topics

Conversational styles – most commonly joking or banter – can be understood as an overlap between care-centred and person-centred work. The adjustment made this an example of empathy that included reasoning about feedback, with the care user as empathiser. During a participant observation, Tilly (woman, 80) joked with the staff who visited her and later said in the follow-up interview that she used different humorous jargon with different staff members. With two female staff members she used to tell dirty jokes, because she knew they appreciated that. She used a different jargon with a young male staff member, joking about his frizzy hair and how it looked as if he had caught his hair in an electric whisk:

Tilly: That’s what I said, that’s what I said today when he came, I said; oh, here comes the electric whisk!

Researcher: Electric whisk? How did he receive it when you joked about it?

Tilly: Well, he likes it; it was he who came up with it.

It is not clear how much staff appreciated Tilly’s jokes, but what is relevant here is that she adapted her conversational style to emphasize the unique character of home care workers, beyond their role as staff members.

There were also instances when a care user adapted to the interests of staff members as unique individuals to facilitate elaborate forms of interaction. This was the case for Hulda (woman, 79) who was granted ‘social time’ with home care workers – an hour every fortnight to talk about her personal interests. Differences in age and background could make it difficult to find common ground for these conversations, and the care theory approach would stress the need for staff to find topics of personal interest to the care user. Hulda’s strategy, however, was to let the home care staff talk about matters of interest to them, thus achieving topical attunement. Hulda described a recent encounter with a staff member of Chilean descent about the political situation in Chile, something Hulda herself was knowledgeable about. This strategy made it possible for the staff member to act as a conversation partner, despite their limited ability to relate to Hulda’s real interests. Hulda described herself as a skilled listener, capable of taking on that role when others talked about matters they cared about.

### Showing an interest in the individual

There are several possible reasons for care users’ interest in the lives of staff members, one being self-interest (Söderberg & Melin Emilsson, 2022) and another – seen in our data – serving to acknowledge individual care workers as people. Showing an interest took several forms. The respondent Frans (man, 91) returned several times to the importance of addressing staff by their correct names. This made them feel better, he said:

I often ask about their name and how it should really be pronounced, and many have abbreviated their names so that people can pronounce them, but I want to know what their real name is and I try to use it and they like to hear their real name.

Many Swedish eldercare staff have non-European backgrounds and it is not uncommon for them to abbreviate their names or use nicknames. By using correct names Frans treated staff members as unique individuals and made the effort to go beyond the ‘working names’ that staff would not use with those who spoke their languages.

Asking about the personal lives of staff was another way of showing interest. Elisabeth (woman, 70), described the life she and her husband lived as monotonous, spending their days in front of the television. Elisabeth had no children of her own and explained in interviews that she was actually not interested in staff members’ families. Nevertheless, she usually asked them about their children and holidays. She knew who had dogs and who had children and could adjust her questions accordingly. With her person-centred work, Elisabeth acknowledged home care workers as individuals, beyond the role of staff members.

Yes, when there are weekends and so, you can ask that; well, ‘Did you have a nice weekend?’ or something. We almost never do anything ourselves because we don’t have the energy. But they have things to talk about. And similarly, when they have been on holiday and the like, we usually ask about that; ‘Well, did you have a nice holiday?’ and ‘Have you been away somewhere?’ and something like that. So of course, we talk about that too. Or those who have children or have a lot of pets, there you can also ask that; what about that kitty or that dog and horse and so on?

These types of questions were frequently mentioned in our interviews. They generally concerned their family situation, pets, homes, and descent as well as recent activities and problems in life. Questions about biographical information were framed as following social conventions – ‘I think one should show some interest in the area they come from’ – and as a route to a more personal contact with staff members. Several respondents suggested that such relationships depended on the personalities of individual care workers, whether or not they tallied with the care user’s personality or if there was common ground.

A common way to show an interest in the lives of home care workers was small talk about health problems. Like Lewinter (2003), we find the concern shown to be a form of reciprocity. A typical instance from the observations was when a care user mentioned their prior knowledge of a home care worker’s health issues. One example was in an observation, when Tilly chatted with staff about the problems a staff member was having with her feet, and in the follow-up interview Tilly noted: ‘I suppose you become, yes, you become intimate with them. It’s really nice, but it’s not with everyone, but these two, they’re good.’ Staff members’ health issues could act as a bridge to talking about private matters since such problems were to some extent work-related, as for example when a member of staff returned from a sick leave.

Some respondents described relations where they regularly offered support or advice to home care staff with special needs. Charlotte was concerned about some younger male care workers, who had migrated to Sweden as unaccompanied minors from Afghanistan and were now working in home care:

Yes, take for example the Afghan refugee children; there are two very cute boys. It’s mostly men, actually, that I have. And they, yes, they can talk about their difficulties, for example that now they cannot, they are not at all sure of getting a residence permit, even though they work and behave well, great at this job and someone is then training for assistant nurse for example. And then they now risk being deported. This country wouldn’t function without them. I think that’s really … yes, and then they can have problems because then someone has sent his entire salary to his mother who needs surgery …

Charlotte said her home had become a place for people to moan and complain, but her concerns were not discussed, only other people’s. She concluded that she was happy if she could be of “some use” and she enjoyed her conversations with the young men from Afghanistan who needed the support she could provide by listening to their grievances, even if it meant there was less time for other tasks.

### Finding common ground

Person-centred work could also be about identifying common denominators, such as having connections to the same city, knowing the same people, or sharing common interests. This form of relational work was closely linked to talking about problems, as seen. Andreas described discovering that he had been in a relationship with a woman known to one of the staff members:

And with that it kind of eased up; we can talk a little about this and that. I mean, she has opened up and told me that she has a daughter who has a bit too many diagnoses and she has always had problems with her. And I mean, you don’t do that very openly otherwise.

A similar background or interests, coming from the same place, knowing the same people: it was a form of community that meant relations could move beyond the formal care arrangements. This aim was explicitly discussed in the interview with Olof (man, 82) and Olivia (woman, 73). The couple had worked in several countries in Africa and the Middle East and described situations where they had told staff from those countries about their life there. Olivia asked her husband if he recalled a care worker from Zimbabwe who had been ‘amazing’, and Olof explained:

And then it is of relevance that we had lived in Harare. And when she found out, (laughs)…that we had lived there right behind the bus station, it kind of became a better contact.

Olof said their experience of foreign countries made for interesting chats and he thought their personalizing approach made it fun for staff to visit their home. His way of moving relations away from the care context could also be regarded as a way of detaching relations from their formal care framework. Olof argued that:

it’s not at all in, in the professional role here, but it’s a bonus, eh, a bonus for me in the first place, eh, a bit of a bonus for them not to be so tied to manuals and, and so.

Identifying common denominators was not only about person-centred relations with staff, but also a type of care-centred work. Talking about common interests and experiences were perceived as something that improved staff members working conditions, by making them feel less controlled by instructions and guidelines. Like Charlotte and several others, Olof described a form of personalized care encounter, as opposed to a standard version that staff were supposed to provide.

The most obvious common denominator in our interviews was gender and shared experiences of being a woman. Discussions between female care users and female staff covered a range of topics, from the menopause to hair products, reflecting shared experiences. Lena, a trained psychologist, illustrated several cases where staff members brought their problems to her. She even encouraged it, since she knew she was in a position to help. On a later occasion, though, it transpired Lena had changed her position, partly due to the frustratingly high staff turnover. Initially, the shared experience of womanhood had enriched Lena’s interactions with female staff, as she felt she could take a supportive approach. However, over time the situation became overwhelming, leading to Lena feeling inundated by home care workers’ personal issues. She commented on the situation with ‘I don’t need to know their menstrual cycle’. The channel for sympathy that Lena had opened had been overused, resulting in emotional fatigue. The situation illustrates the risks of staff sharing too much of their private lives, making care users’ relational work a burden.

## Conclusions

Our study adds to the literature on formal care by drawing attention to the relational work that is continually carried out by care users. While research about care shows that emotion work like the use of empathy has a relational character, studies have rarely gone beyond the basic notion of carer as empathiser and care as empathee (Van Dijke et al., 2019). Our study shows that care users frequently adopt the role of empathiser and sympathizer.

One outcome of the study is the realization that older care users’ efforts may be deliberately aimed at improving the provision of care and the conditions for care staff by boosting their confidence, showing sympathy, and avoiding potentially embarrassing situations. Additionally, care users establish personal relations that go beyond the immediate provision of care, making staff members visible as individuals, and offering sympathy and support. While these activities may be seen as ways to increase the wellbeing of older care users themselves, it is reasonable to conclude that care users are involved in a form of relational work to benefit those who provide care, like the rationality of caring described by Wærness (1984), similar to the type of recognition of the individual that Fazio et al. (2018) argue is central to person-centred care.

As noted by Fine (2015), little attention has been paid to older care users as active agents, unlike the situation in disability studies. Given the number of care users in different care regimes, this is unsatisfactory. The relative lack of interest could even be regarded as a form of ageism as it keeps older people’s perspectives out of sight. Therefore it is relevant to propose a research programme about care use and the development of theory that proceeds from the findings of our study. When outlining such a programme, phenomena identified in studies of formal carers are a fruitful place to start. Strandell (2022) and Elmersjö et al. (2022) investigate moral stress and the ways staff in eldercare compensate for a lack of time to provide good care. It is relevant to explore moral stress among care users and what strategies they use to counteract it. Other avenues include attempts to establish control over the home and body (Dyck et al., 2005), and the choice of preferred carers alongside the degree of personal engagement in the lives of staff who care users appreciate, including cases where relations take problematic directions (Ahnlund et al., 2023). There is also a need to dig deeper where several of our respondents said the same thing: there were staff members who acted outside their professional role, and who were appreciated for the personal contact they allowed to develop. Some care users stated that activities and contact arrangements they appreciated were actually prohibited and should be kept secret. It is easy to see why these practices might be ethical challenges for the provision of formal care.

One argument for the proposed programme is the very existence of these activities among care users. It certainly warrants being identified and studied in detail as part of formal care. A second argument concerns the micro politics of care. Most respondents talked about aspects that could be called care-centred work or person-centred work, but some seemed especially capable of ‘handling’ relations with staff, based on their personalities, professional background, education, or other experience. Studies of care users’ relational work offer a way to understand care inequalities and increase awareness of the dynamics and characteristics – class, gender, ethnicity, cultural capital – that leave some care users better equipped to succeed in their relational work. Is it the case that some care users develop skills over time? A third argument for pursuing further studies concerns the context that comprises the care workers’ ‘working conditions’. Swedish home care users are likely to encounter many individual staff members in even a short timeframe. Some staff members will remain at the organization for an extended time, but many may change jobs within just a few months. Care users are forced to handle situations where new staff members enter their home, and they must be wary of overinvesting emotionally in the people they grow fond of. The emotion work done by Swedish home care users can be seen both as a practice intended to strengthen their relations with specific staff members and as a practice aimed at handling short-term acquaintances and a lack of time for the care work that has been granted. Our study demonstrates that care users displayed – and, like Hulda and Janet, reflected on – competence in care use governed by their sympathies for the care workers’ working conditions they encountered, a finding that is echoed in studies of care workers (Strandell, 2022). By performing emotion work designed to put staff members at ease who would otherwise experience an even more stressful, unpleasant working situation, care users make up for insufficient resources allocated to the home care sector. It is possible some care users are more successful while others largely fail at achieving a ‘satisfactory’ competence, adapted to the current care regime, by protesting when services are performed poorly. Studies of care use may reveal that the regime competence fostered by the organization of formal care ultimately results in adaptive behaviour, while protest may be perceived as a lack of empathy and a failure to collaborate. These dynamics are likely to appear as regime-dependent in other countries too.

Our study has several implications for practice. Recognizing that many care users act as empathizers and sympathizers can counter the kind of dehumanization that may occur in care relationships. The study sees the care user as a person with a particular agency, which may add a new perspective to the personalization approach. The complex issue of what relationships mean, beyond the professional encounter, also needs to be discussed in a care organization context. Many care users seem to appreciate care staff talking about their personal problems and building friendships; however, we establish that staff need to find a balance so talking about private problems do not burden the care user who is listening with relational work. To be personal but not private is often said to be the ideal for staff in caregiving relations. Care organizations can hardly encourage staff to talk about private problems at work. Yet, interactions and relations of this kind are an important factor in care use, and it is also doubtful to what extent it should be prohibited. This study shows that staff should not only consider their own relational work, but also recognize that older people are engaged in reciprocal relational work with them.

## Limitations

A limitation of the study is that the voice of care staff is absent and, although several participant observations were conducted, most descriptions and conclusions on relational work among care users are based on what they said when interviewed.
